# Discovery and Cardioprotective Effects of the First Non-Peptide Agonists of the G Protein-Coupled Prokineticin Receptor-1

**DOI:** 10.1371/journal.pone.0121027

**Published:** 2015-04-01

**Authors:** Adeline Gasser, Simone Brogi, Kyoji Urayama, Toshishide Nishi, Hitoshi Kurose, Andrea Tafi, Nigel Ribeiro, Laurent Désaubry, Canan G. Nebigil

**Affiliations:** 1 Biotechnology and Cell Signaling Laboratory (UMR 7242), CNRS-University of Strasbourg, Illkirch, France; 2 European Research Centre for Drug Discovery and Development (NatSynDrugs), University of Siena, Siena, Italy; 3 Departments of Biotechnology, Chemistry and Pharmacy, University of Siena, Siena, Italy; 4 Department of Pharmacology and Toxicology, Graduate School of Pharmaceutical Sciences, Kyushu University, Fukuoka, 812–8582, Japan; 5 Therapeutic Innovation Laboratory (UMR7200), CNRS-University of Strasbourg, Illkirch, France; Emory University, UNITED STATES

## Abstract

Prokineticins are angiogenic hormones that activate two G protein-coupled receptors: PKR1 and PKR2. PKR1 has emerged as a critical mediator of cardiovascular homeostasis and cardioprotection. Identification of non-peptide PKR1 agonists that contribute to myocardial repair and collateral vessel growth hold promises for treatment of heart diseases. Through a combination of *in silico* studies, medicinal chemistry, and pharmacological profiling approaches, we designed, synthesized, and characterized the first PKR1 agonists, demonstrating their cardioprotective activity against myocardial infarction (MI) in mice. Based on high throughput docking protocol, 250,000 compounds were computationally screened for putative PKR1 agonistic activity, using a homology model, and 10 virtual hits were pharmacologically evaluated. One hit internalizes PKR1, increases calcium release and activates ERK and Akt kinases. Among the 30 derivatives of the hit compound, the most potent derivative, IS20, was confirmed for its selectivity and specificity through genetic gain- and loss-of-function of PKR1. Importantly, IS20 prevented cardiac lesion formation and improved cardiac function after MI in mice, promoting proliferation of cardiac progenitor cells and neovasculogenesis. The preclinical investigation of the first PKR1 agonists provides a novel approach to promote cardiac neovasculogenesis after MI.

## Introduction

Cardiovascular disease is the leading cause of morbidity and mortality worldwide [1]. Heart failure causes the death of an estimated 17.1 million people each year. According to World Health Organization estimates, almost 30 million people are expected to die from heart disease and heart failure by the year 2020. New strategies are urgently required for the activation of cardiomyocytes survival signaling and the promotion of neovasculogenesis, increasing the number of progenitor cells for the treatment of heart failure.

Prokineticin-1 and 2 are potent angiogenic and anorexigenic factors that use two G-protein coupled receptors (GPCRs); PKR1 and PKR2 [2,3]. Prokineticin-2 is the most potent agonist of both receptors [2]. PKR2 is the dominant receptor in the adult brain, particularly in the hypothalamus, the olfactory ventricular regions, and the limbic system, whereas PKR1 is widely distributed in the periphery [4]. These receptors are coupled to G_αq_, G_αi_ and G_αs_, mediating intracellular calcium mobilization, the activation of MAPK and Akt kinases, and cAMP accumulation, respectively [5].

We recently demonstrated that PKR1 signaling protects cardiomyocytes against hypoxic challenge, by activating the PI3/Akt pathway [6]. Prokineticin-2 induces significant outgrowth from mouse epicardial explants and quiescent epicardium-derived progenitor cells (EPDCs), restoring their pluripotency and triggering the differentiation of endothelial and vascular smooth muscle cells. These effects of prokineticin-2 were abolished in EPDCs derived from PKR1-knockout (KO) hearts, demonstrating the involvement of PKR1. Transient PKR1 gene transfer after coronary ligation in the mouse model of myocardial infarction (MI) reduced mortality and preserved heart function by promoting cardiac angiogenesis, cardiomyocytes survival and the proliferation of EPDCs [7]. PKR1 and PKR2 are 85% identical and both are expressed in cardiovascular tissues [6]. However, the signaling pathways mediated by PKR1 and PKR2 act in opposite directions in the postnatal heart [8, 9]. PKR1 signaling in endothelial cells promotes proliferation, migration and angiogenesis [10]. By contrast, PKR2 is coupled to the G_α12_ signaling pathway and downregulates the adhesion molecule ZO-1, leading to endothelial cell disorganization and fenestration. PKR2 has detrimental effects on the heart, inducing cardiac hypertrophy and vascular leakage. PKR1 signaling was shown to have beneficial effects, using transient PKR1 gene transfer after coronary ligation in the mouse model of MI [6]. This suggests that PKR1 is a potential novel treatment target for limiting myocardial injury following ischemic events [6].

A prokineticin-2β peptide containing only 47 amino acids from the N-terminus of prokineticin-2 has been reported to be a potent and selective PKR1 agonist [11]. However, the use of peptide drugs is hampered by their intrinsic metabolic instability and low bioavailability. Peptide drugs have been administered by the parenteral route, because of *their extensive degradation in* the gastrointestinal tract. However, non-peptide PKR1 agonists have never before been reported yet, despite the discovery of several PKR antagonists [12, 13, 14, 15, 16]. Indeed, the discovery of non-peptide agonists of GPCRs is much more challenging than that of antagonists: more than 500 non-peptide ligands for over 70 peptidergic GPCRs have been identified, but most of these drugs are antagonists [17].

Molecular modeling plays a key role in GPCR ligand discovery, because most GPCR structures remain unsolved. Accordingly, computational techniques, such as homology modeling and *in silico* screening (high-throughput docking), have proved useful for GPCR drug discovery [18, 19, 20, 21, 22]. We aimed to identify novel small molecules capable of protecting the heart against MI by screening *in silico* of a large chemical 3D chemical database using high-throughput docking (HTD) technique. We identified and validated IS20 as a selective PKR1 agonist that strongly protected heart function in a mice model of myocardial infarction (MI). This study thus provides proof-of-concept for the development of selective non-peptide PKR1 agonists for the treatment of heart failure.

## Materials and Methods

### Ethics statement

The animal studies were approved by the Animal Care and Use, and ethics committees of the Bas-Rhin Prefecture (Permit Number: B67-274) with the recommendations in the Guide for the Care and Use of Laboratory Animals of the French Animal Care Committee, with European regulation-approved protocols. The animal experimentation and housing were conducted at the accredited animal experimentation and housing facility of the Institut de recherche de l’Ecole de biotechnologie de Strasbourg (Register number: C67-218-19).

### Animals

Male C57BL/6J mice (18–20 g) aged 10 to 12 weeks were maintained under a 12 h light-12 h dark cycle, with free access to food and water. They were obtained from Charles River Laboratories (L’Arbresle, France) and Janvier (Le Genest-S^t^-Isle, France). All animal experiments were carried out in accordance with current institutional guidelines for the care and use of experimental animals. All efforts were made to minimize suffering with respect to the regulation concerning genetically manipulation of organisms.

### Culture of ECs and assays of the formation of tube-like structures

H5V endothelial cells derived from mouse heart were kindly provided by Dr. Annunciata Vecchi (Istituto Clinico Humanitas, Rozzano, Italy). H5V cells were infected by 1 MOI of adenovirus carrying PKR1 cDNA as previously described [10]. The cells were cultured in DMEM supplemented with Glutamax, 4.5 g/l glucose and 10% fetal calf serum, non-essential amino acids, 2 mM glutamine, 10 μg/ml heparin, and 100 μg/ml endothelial cell growth supplements (ECGS) (Tebu-Bio). We coated 24-well culture plates with Matrigel (BD Biosciences), according to the manufacturer's instructions. H5V cells were harvested by trypsin digestion and used to seed the coated plates at a density of 10^5^ cells per well, in the serum-free assay medium with or without prokineticin-2 (10 nM). The compounds were dissolved in DMSO (10 mM stock solution) and diluted in cell culture medium. Corresponding dilution of DMSO was used as vehicle for each experiment. The plates were then incubated at 37°C for 24 h. The formation of two-dimensional branched tube-like structures was observed with an inverted phase-contrast microscope (Leica), and the number of branching points was determined for five random fields from each sample [10]. Each experiment was carried out four times. Images were captured at 10× magnifications, with a microscope fitted with a digital photography system.

### Internalization assay

CHO cells stably expressing a construct encoding PKR1 fused to EGFP were used to seed glass coverslips coated with polylysine (WI; 0.01%; Sigma-Aldrich, St. Quentin, France) to 20% confluence. Internalization experiments were performed by incubating the cells at 37°C for various times with 10^−7^ M prokineticin or 10^−6^ M IS1, as previously described [23]. Cells were then mounted in Mowiol for confocal microscopy analysis. Ligand-induced PKR1 receptor-EGFP internalization was then quantified by confocal microscopy coupled to digital image analysis, as previously described [23]. EGFP fluorescence was detected following excitation at 488 nm by use of a spectrophotometer set with a window between 530 and 600 nm. Each image (1024 × 1024 pixels, ×63 oil-immersion objective) was done on a cross-section through the cells. Image J software allowed to measure the mean density of the surface (S), cytoplasm (C), and nucleus (N) fluorescence. The background fluorescence (N) was subtracted from the S and C values. The S/C ratio provides reliable information about the level of cell surface expression and the internalization state of the GFP-PKR1.

### Membrane preparations and radioligand binding experiments

Membrane preparations from CHO cells stably expressing the wild-type human PKR1 receptor were prepared as previously described [13]. The cells were transfected with 1 μg of required plasmids (hPKR1) using Lipofectamine reagent 2000 (Invitrogen). CHO cells stably expressing PKR1 (2.3 pmol/mg protein) were maintained in MEM (Gibco) supplemented with 10% foetal calf serum, 2mM L-glutamine and 2.5 μg/ml fungizone, 400 μg/ml G418 and 300 U/ml hygromycin. These cells (1–5 μg total mass of membranes/assay) were incubated for 1 h at 20°C with 2 × 10^−10^ M [^125^I]-MIT (PerkinElmer) in binding buffer alone or in the presence of various concentrations of prokineticin-2 (Peprotech) or IS1. The reaction was stopped and the mixture was passed through Whatman GF/C filters. The filters were then washed and their radioactivity was counted. Saturation-binding curves were obtained by incubating membrane proteins with various concentrations of [^125^I]-MIT. The competitive binding profiles were analyzed by nonlinear regression analysis, with PRISM 3.02 (GraphPad Software).

### Confocal microscopy-based calcium release analyses

For [Ca^2+^]_I_ measurements in cell monolayers, we incubated the cells for 40 minutes at room temperature with 5 M fluo-4/AM (Molecular Probes). CHO cells expressing PKR1 were imaged with a Zeiss LSM-410 inverted confocal microscope (Carl Zeiss, Inc., Germany) [24]. Changes in cytosolic Ca^2+^ were measured by converting the 340/380 ratio of fluorescence (after background subtraction) using the method of Grynkiewicz [25]. All imaging data were obtained in line scan mode, generally with the scan line oriented along the long axis of the cells, avoiding cell nuclei. Each image consisted of 512 line scans obtained at 2.09 ms intervals, each comprising 512 pixels 0.156 *μ*m apart. We used a Zeiss Plan-Neofluar 40× oil immersion N.A. = 1.3 objective and the confocal pinhole was set as recommended by the manufacturer, to obtain a spatial resolution of 0.4 *μ*m in the horizontal plane and 0.9 *μ*m in the axial direction. Images were processed and the data were analyzed and presented with IDL software (Research Systems, Boulder, CO). Experiments were carried out at room temperature (20–22°C).

### Kinase assays on hearts and cells

Animals were anesthetized with 1% pentobarbital, followed by an intraperitoneal (i.p.) injection of IS20 (0.5 mg/kg). The mice were sacrificed 10 or 20 minutes after the injection and tissues were harvested for protein extraction [26]. Tissue samples were homogenized in a lysis buffer consisting of 50 mM Tris-HCl pH 6.8, 1 mM EDTA pH 8.0, 1% NP-40, 1 mM Na_3_VO_4_, 0.1% SDS, 100 mM NaCl, and phosphatase and protease inhibitors. Homogenized samples or lysed CHO cell samples were incubated for 1 h at 4°C, with shaking, and were then centrifuged at 13,000 rpm and 4°C to obtain protein extracts. Protein concentration was determined with the BCA assay (Thermo Scientific), according to the manufacturer’s instructions. About 50 μg of protein for each sample was subjected to SDS-PAGE in a 12% polyacrylamide gel. The resulting bands were transferred to a PVDF membrane, which was incubated with anti-phospho-Akt serine 473 or anti-phospho-ERK antibodies (both at a dilution of 1:1000; anti-Akt antibody supplied by Santa Cruz and anti-ERK by Cell Signaling). Samples were treated with peroxidase-conjugated secondary antibody (Santa-Cruz; 1:5000 dilution), and immunoreactivity was detected with an ECL chemiluminescence detection kit, according to the manufacturer’s instruction (Amersham Pharmacia). The intensity of the resulting bands was determined with Image J software.

### Animals, surgical procedures, echocardiography and blood pressure assessment

We caused MI in 10- to 12-week-old male C57BL/6J mice (weighing 20–25 g, *n* = 20 each group), by ligating the left anterior descending coronary artery following isoflurane anesthesia, as previously described [6]. Systolic function was assessed in 24-week-old male mice (*n* = 6 for each group), by echocardiography in M-mode (Sonos 5500, Hewlett-Packard, with a 15 MHz linear transducer), with two-dimensional measurements [9]. We calculated the mean measurements for six selected cardiac cycles for at least two independent scans performed in a randomized, blind fashion, with papillary muscles used as reference point to ensure that all scans were carried out at the same level.

### TUNEL and BrdU assays

TUNEL (terminal dUTP nick end-labeling) assays were performed with an *in situ* cell-death detection kit (Roche Diagnostics GmbH, Mannheim, Germany), according to the manufacturer’s instructions [6]. Mouse heart samples were obtained three days after coronary ligation. For BrdU staining, mice were treated with 100 μl BrdU (20 μg/ml, i.p.) three days after MI. Four hours later, their hearts were excised for immunostaining experiments. We analyzed 20 different heart sections (*n* = 3 animals for each group) from the zone bordering the ischemic area, by fluorescence microscopy. Slides were counterstained with DAPI. We acquired 6 to 10 images per heart (*n* = 3 animals) with a fluorescence microscope (Leica). The TUNEL labeling index was calculated as the mean number of DAPI-stained TUNEL-positive nuclei on 10 high-power microscopic fields (x 40) per heart.

### Computational Details

The computational details are shown as supplementary experimental procedure in S1 File.

### Data and statistical analysis

Values are expressed as means ± SEM. One-way ANOVA or ANOVA for repeated measures followed by Fisher’s protected least significance test or by Student’s unpaired *t* test (peptide *vs*. vehicle) was used to assess the significance of differences.

## Results

### Homology modeling and high-throughput docking

For the identification of PKR1 agonists, we built a 3D model of PKR1 by homology modeling approach. The model of PKR1 was generated with CPHmodels 2.0 [27], a web server application for fold recognition/homology modeling. The amino-acid sequence of human PKR1, in fasta format, was obtained from UniProtKB (entry Q8TCW9; 393 aa) and submitted to CPHmodels 2.0 [28]. The model was refined and optimized in the Maestro software suite [29, 30, 31]. Analyses of the predicted PKR1 extracellular binding site revealed the existence of a putative active site, located essentially in the transmembrane domain and involving interactions between helices III, V, VI and VII, with the marginal involvement of helix II, and corresponding to a potential allosteric site for PKR1. Comparable extracellular binding site has been already reported for β-adrenergic receptors and adenosine A_2a_ receptors, based on their crystal structures as discussed below [32, 33, 34, 35].

**Fig 1:**
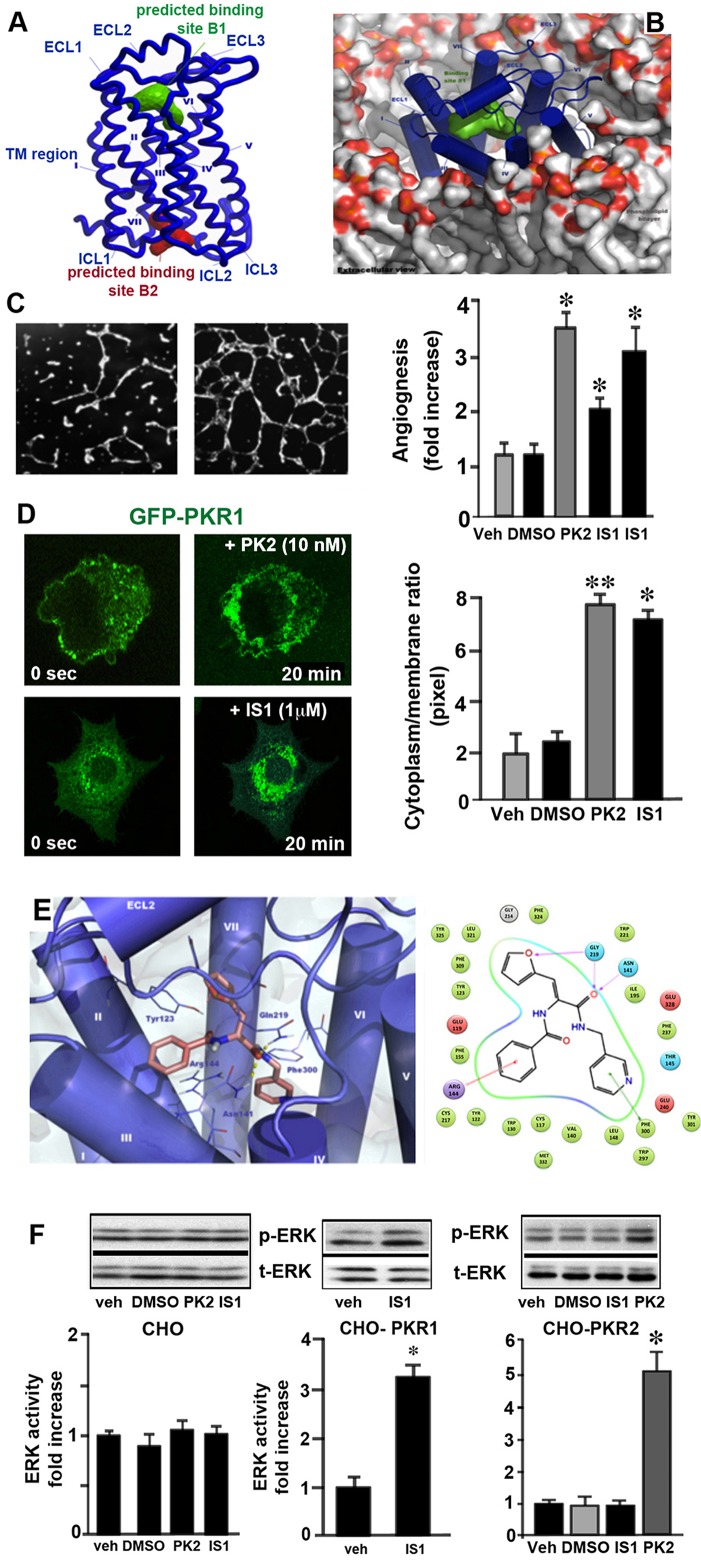
PKR1 homology model predictions of binding sites and evaluation of IS1 pharmacology and its interactions with PKR1 binding sites. **A.** Illustration of the extracellular loop (ECL), intracellular loop (ICL) and transmembrane (TM) regions of PKR1. Output of ICM Pocket Finder: the first binding site, shown in green, is located on the extracellular side of PKR1, whereas the second (in red) is located on the cytoplasmic side. **B.** Extracellular view of PKR1 on the cell membrane highlighting the predicted binding site used for HTD (green solid surface). **C.** Representative illustration showing that vehicle-treated endothelial cell cannot induce the formation of tube-like structures (*in vitro* angiogenesis). However, the PKR1 agonist IS1 induces the formation of tube-like structures in CHO cells transfected with PKR1. IS20 promotes intracellular calcium release, as detected by measuring Fluo-4AM intensity by confocal microscopy, within 20 minutes. The histogram shows the % angiogenesis induced by prokineticin-2 (10 nM) and IS1 (1 and 10 μM). **D.** Representative illustration of the situation 0 and 20 minutes after the treatment of CHO cells expressing PKR1-EGFP with IS1. IS1 causes the internalization of PKR1-EGFP within 20 minutes. The histogram shows the internalization of PKR1 induced by prokineticin-2 and IS1 as a cytoplasm/ membrane (C/M) ratio. **E.** Docked pose of IS1 (light pink sticks) in the PKR1 homology model-(blue cartoon) binding site. The yellow dotted lines indicate hydrogen bonds. Key interactions of IS1 with PKR1 binding sites are shown as follows: the green lines indicate π-π stacking, the red lines cation-π stacking and the purple lines hydrogen bonds. **F.** IS1 (100 nM) cannot increase ERK activity in the absence of PKR1 in CHO cells. However, IS1 activates ERK kinase in the presence of PKR1 in CHO cells. IS1 does not activate ERK kinase in CHO cells expressing PKR2 unlike prokineticine-2 (PK-2). * p <0.05, versus values from vehicle by Student’s *t*-test.

For HTD, PKR1 was submitted to ICM Pocket Finder for the identification of potential binding sites. Two potential binding sites were found (Fig 1A). The first is located on the extracellular side of the receptor (green in Fig 1B; volume of 264 Å^3^), whereas the second is on the cytoplasmic side of the receptor (red in Fig 1A; volume of 211 Å^3^). Similar results were recently reported in a PKR modeling study [36]. A predictive analysis of PKR1 extracellular binding sites revealed the existence of a putative active site, located principally in the transmembrane domain and involving helices III, V, VI and VII, with a small role for helix II, corresponding to a potential allosteric site for PKR1. Transmembrane allosteric binding sites have already been described for β-adrenergic receptors and adenosine A_2a_ receptors, on the basis of their crystal structures [32, 33, 34, 35].

The extracellular binding site identified in the PKR1 homology model was used for HTD to identify small molecules that would be expected to bind to the predicted active site of PKR1. HTD was carried out with GOLD 3.0.1 software and a commercially library of about 250,000 small molecules (Asinex Gold Collection; Asinex Ltd, Russia). We used the scoring function ChemScore [37] to rank compounds best fitting the identified binding site. The selection was based on the use of a ChemScore cutoff of 40 coupled with cluster analysis and visual inspection. Following this protocol, we selected 10 potential PKR1 ligands based on the unique cluster of docked solutions. These ligands were then purchased (Asinex Ltd, Russia) and subjected to biological evaluation.

### Biological testing of virtual screening hits

PKR1 receptor signaling has been shown to promote the formation of tube-like structures of coronary endothelial cells (H5V) infected with an adenovirus carrying PKR1 cDNA on Matrigel, as an *in vitro* model of angiogenesis [10]. Growth in Matrigel leads to the differentiation of many cell types and induces the formation and organization of endothelial cells into capillary tubules. We used this assay to compare the 10 structurally different compounds with prokineticin-2, in terms of their capacity to promote angiogenesis. One of the 10 putative ligands, IS1, significantly induced angiogenesis *in vitro* when added to a concentration of 10μM dose. This effect was similar to that of prokineticin-2 (Fig 1C). We also examined whether the induction of angiogenesis by IS1 was mediated by PKR1, by adding this compound to H5V cells in the absence of PKR1. Similarly to prokineticin-2, IS1 did not induce the formation of tube-like structures in the absence of this receptor.

### Capacity of IS1 to induce PKR1 receptor internalization

We tested the receptor-binding properties of IS1 by measuring its ability to internalize GFP-coupled PKR1 in CHO cells. Confocal microscopy analysis of CHO cells stably expressing the human PKR1 receptor fused to EGFP in resting conditions revealed intense PKR1-EGFP fluorescence at the plasma membrane and in the cytoplasm. Incubation with 1μM IS1 for 20 minutes abolished the GFP signaling at the plasma membrane and led to the accumulation of GFP around the nucleus. Quantification of the cytosol/membrane (C/M) fluorescence ratios induced by 1μM IS1 (*n* = 10) or 10nM prokineticin-2 (*n* = 5) showed that maximal internalization values (20 min) were similar for IS1 and prokineticin-2 (Fig 1D and histogram), thereby demonstrating the ability of IS1 to bind and activate PKR1.

Based on the results of the docking protocol, we herein propose a consistent binding mode of IS1 to the receptor. The output of the molecular docking calculation is shown in Fig 1E. IS1 strongly interacts with PKR1 through its aromatic systems, and with Arg144^3.32^ and Phe300^6.51^ by cation-π and π-π stacking, respectively, whereas the oxygen atom of its furyl moiety forms a hydrogen bond with the backbone of Gln219 (Fig 1E, right). Moreover, a series of hydrogen bonds forms between a carbonyl group of IS1 and the extracellular loop residues Gln219 as well as the transmembrane residue Asn141^3.29^. The highly favorable ChemScore (40.44) suggests a high affinity of IS1 for the receptor (where applicable, Ballesteros-Weinstein designations are shown in superscript).

We then investigated the ability of IS1 to compete with radiolabeled ^125^I-MIT, a standard ligand for the PKR1 receptor, in a receptor-binding assay. ^125^I-MIT bound to the PKR1 receptor with high affinity, with a *K*_i_ for the receptor of 50 pM. Prokineticin-2 displaced this ligand with an IC_50_ of 36pM. Interestingly, IS1 could not displace this ligand, indicating that prokineticin-2 and IS1 interact with PKR1 at different binding sites (S1A Fig). Consequently, IS1 enhances functional response of prokineticin-2 on ERK activity (S1B Fig). Moreover, IS1 (100 nM) cannot increase ERK activity in the absence of PKR1, indicating involvement of PKR1 in ERK activity (Fig 1F). However, only prokineticin-2 (10 nM) was able to activate ERK kinase via PKR2 in the CHO cells expressing PKR2 (Fig 1F, right). These data confirm that IS1 is a selective agonist of PKR1.

### Effects of IS1 on PKR1 signaling

**Fig 2:**
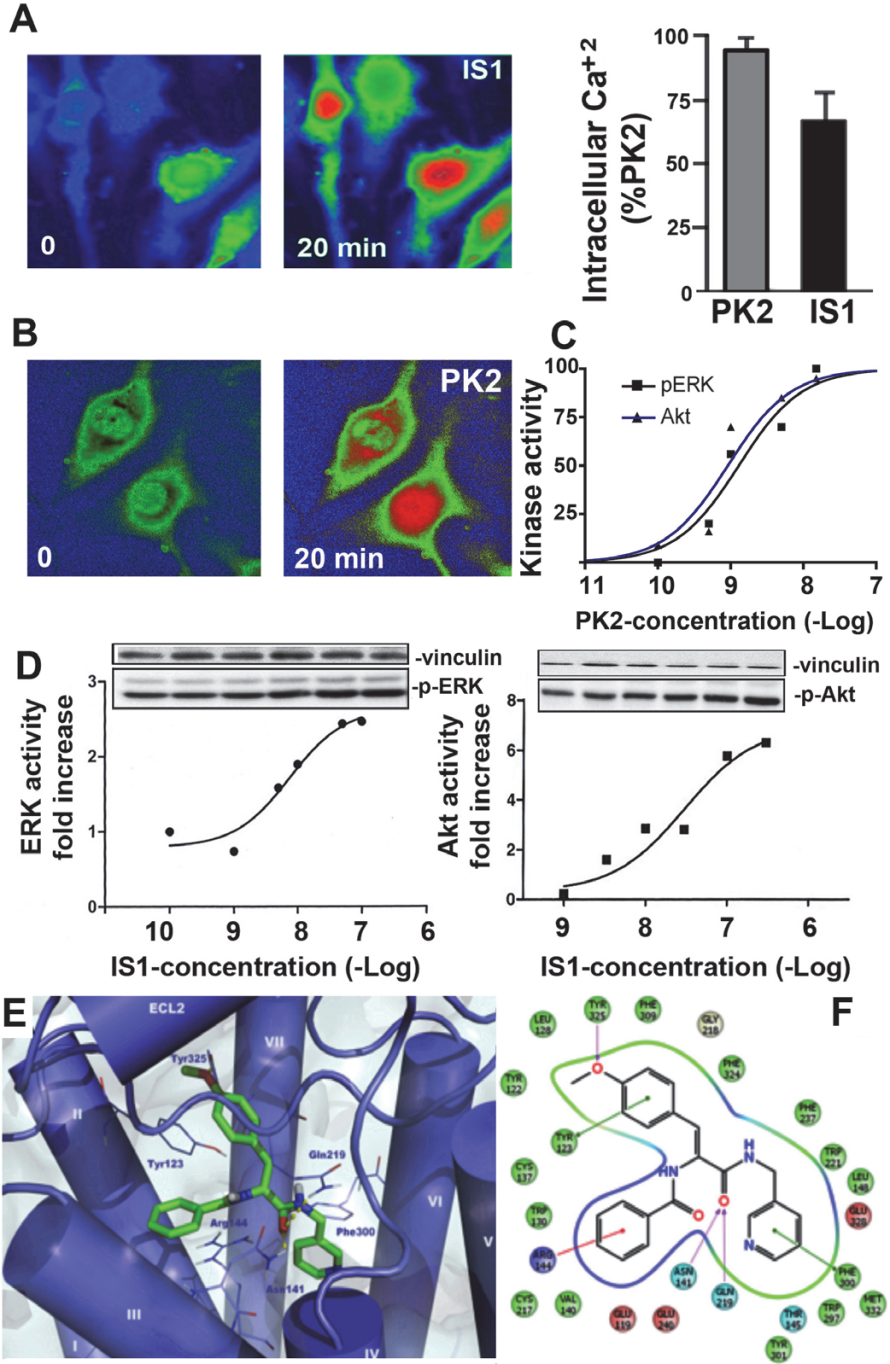
Evaluation of the effects of the non-peptide PKR1 agonist IS1 on PKR1 signaling and docking results for IS20 in the PKR1 homology model-binding site. **A.** Representative illustration of the situation 0 and 20 minutes after IS1 treatment. Calcium was released (red) from intracellular pools of CHO cells transfected with PKR1. IS20 promotes intracellular calcium release, as detected by determining the intensity of the Fluo-4AM signal by confocal microscopy, within 20 minutes. **B.** Histogram showing changes in intracellular calcium concentration induced by IS1 in 20 minutes, as a % of the change induced by prokineticin. **C.** IS1 activates ERK in a dose-dependent manner. **D.** IS1 activates Akt kinase in a dose-dependent manner. **E.** Docked pose of IS20 (green sticks) in the PKR1 homology model-(blue cartoon) binding site. Hydrogen bonds are shown as yellow dotted lines. The molecular docking analysis shows the key interactions of IS20 with the PKR1 binding site. The green lines indicate π-π stacking, the red lines cation-π stacking and the purple lines hydrogen bonds. Data are expressed as the mean ± SEM. * shows p<0.05 versus values from vehicle by Student’s *t*-test.

We explored the effect of IS1 on PKR1 signaling pathway by analyzing its effects on calcium mobilization in PKR1-expressing CHO cells using confocal microscopy. At a concentration of 1 μM, IS1 and PK2 strongly induced calcium release (Fig 2A and 2B). Prokineticin-2 has been reported to activate ERK and Akt kinases in endothelial cells [38, 39, 40]. Similarly, prokineticin-2 activated ERK and Akt in the CHO cells expressing PKR1 (Fig 2C). We therefore also investigated whether IS1 can also promote ERK phosphorylation in these cells. The incubation of CHO cells stably expressing PKR1 with IS1 (10^−8^ to 10^−4^ M) resulted in the concentration-dependent phosphorylation of ERK kinase, with a pEC_50_ of 5.6 ± 0.07 demonstrating the activation of the PKR1 signaling pathway by IS1 (Fig 2D). IS1 (at concentrations of 10^−8^ to 10^−4^ M) also activated Akt kinase by phosphorylation (Fig 2D).

In addition to acting as a potent PKR1 agonist, IS1 has several attractive physicochemical properties and was therefore considered an excellent candidate for hit-to-lead optimization studies.

### Structure–activity relationship and compound optimization

**Fig 3:**
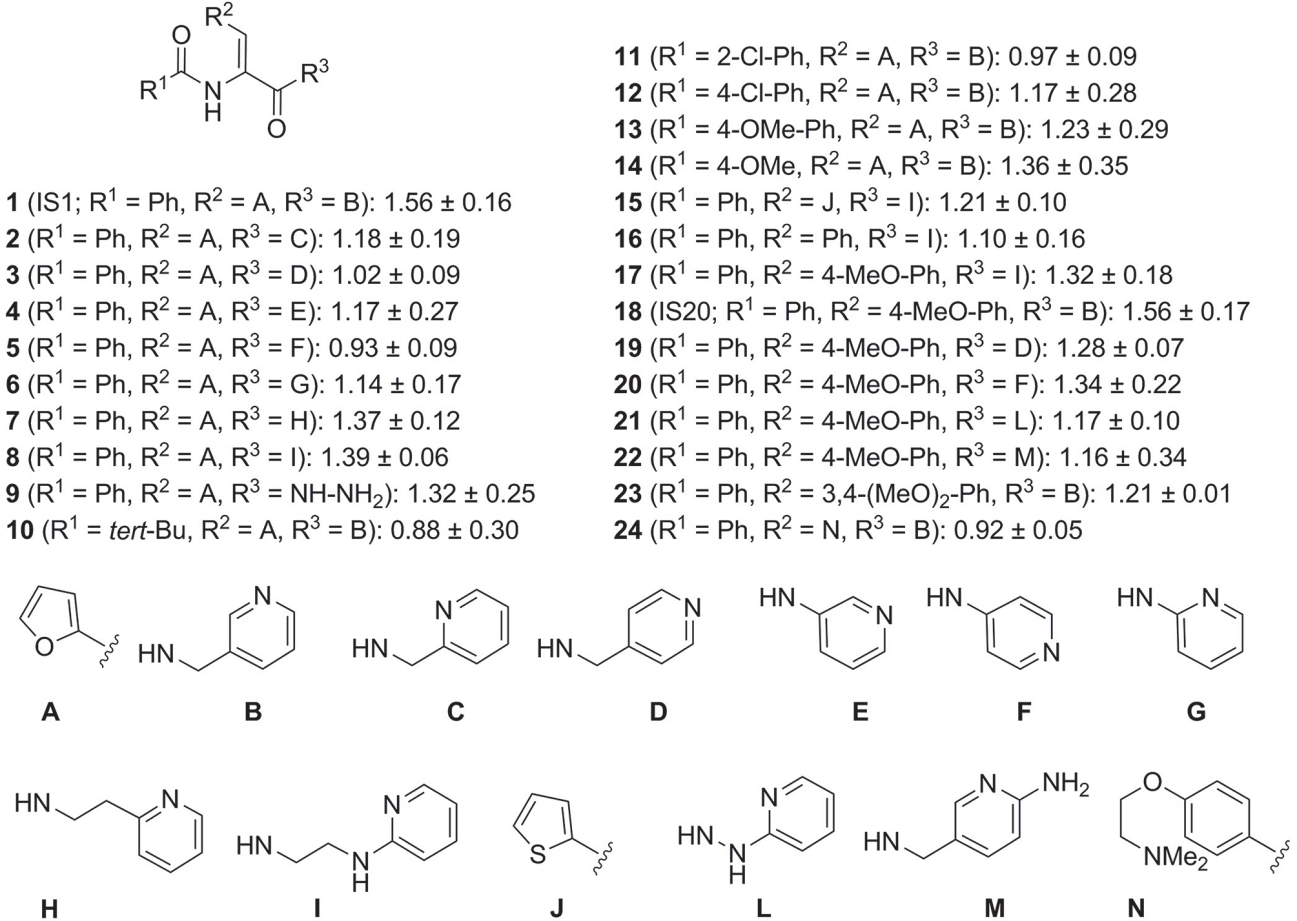
*In vitro* pro-angiogenic activity on endothelial cell (matrigel) at 100 nM expressed as a fold increase over vehicle of IS1 analogues. In this assay, prokineticin-2 (10 nM) used as a positive control induced a 1.48 ± 0.11 fold increase of angiogenesis over vehicle.

IS1 analogues were synthesized as described in the supporting information (S2 Fig) and their ability to induce angiogenesis *in vitro* was analyzed at a single concentration (100 nM). Prokineticin-2 was used as the reference standard (Fig 3). We first explored the effect of changes to the 3-picolylamino moieties. Displacement of the nitrogen on the pyridine moiety (compounds **2** and **3**) and deletion of the methylene from this ring (**4**, **5**) greatly decreased, or even abolished angiogenic activity. By contrast, increases in the distance between the pyridine ring and the amide were well tolerated (**7**, **8**). Surprisingly, the replacement of the *N*-picolylamide by a hydrazide (**9**) was also well tolerated.

We then investigated the effects of substitutions on the *N*-acyl moiety of the dehydroaminoamide core. Replacement of the benzoyl by a non-aromatic pivaloyl group (**10**) abolished activity. The introduction of a chlorine or methoxy group into the benzoyl group in position 2 or 4 (**11**, **12**, **13**) was highly detrimental, whereas incorporating a methoxy group in position 3 was better tolerated (**14**).

Finally, we replaced the 2-furyl groups with other aromatic moieties. The incorporation of a phenyl group was more detrimental than that of a 2-thiophenyl group (compare **16** and **15** with **8**). Conversely, the introduction of a 4-methoxyphenyl group maintained angiogenic activity well (compare **17** and **18** with **8** and IS1 (**1**), respectively). As previously described, the preferred substituent was the 3-picolyl groups (**18**; IS20). The addition of a second methoxy group in position 3 was detrimental **23**, whereas the introduction of a short protonatable amine **24** eliminated angiogenic activity altogether (Fig 3).

The putative binding mode of IS20, as determined by the molecular docking procedure is shown in Fig 2E. As expected, the binding mode of IS20 was very similar to that of IS1 (Fig 2E). However, the replacement of the furyl moiety with a 4-methoxyphenyl group resulted in additional contacts with the binding site not observed for IS1. Indeed, this substituent establishes π-π stacking between its aromatic portion and Tyr123^2.65^, whereas the oxygen of the methoxyl moiety forms a hydrogen bond with Tyr325^7.36^. This substitution improves the ChemScore (42.90) fitness function, suggesting a strong affinity for the receptor, similar to that reported for IS1, as demonstrated by biological assessment.

Both IS1 and IS20 were submitted to QikProp, to obtain a complete picture of the physicochemical properties of relevance for potential drug candidates (S3 Fig). Indeed, for both compounds, all the values obtained were predictive of drug-likeness.

### IS20 is a selective ligand of PKR1 that activates ERK and Akt

IS1 and IS20 had identical activities *in vitro*, but, given that furan derivatives are intrinsically hepatotoxic, we chose to use IS20 for further investigations. We characterized the selectivity of IS20, by assessing its ability to activate ERK kinases in the absence of PKR1 or in the presence of PKR2, in CHO cells (S4A Fig). This compound did not increase ERK activity in CHO cells without PKR1 or in CHO cells transfected with a plasmid encoding other receptor PKR2. Indeed, IS20 was not able to induce angiogenesis in endothelial cells without PKR1. These findings suggest that ERK activation and angiogenesis in response to IS20 require PKR1 receptors but not PKR2, similarly to IS1. We then investigated the ability of IS20 to activate Akt phosphorylation in CHO cells expressing the PKR1 receptor. We obtained EC_50_ values of 44nM ± 0.5 (*n* = 3) (S4C Fig). Moreover, in the presence of IS20 (1nM), PK2 (EC_20_ values of 1nM) mediated ERK activity was dramatically elevated. In combination with an orthosteric agonist PK2, IS20 enhanced the functional response of the PK2 (S4B Fig). These data show that IS20 acts as a positive allosteric modulator. IS20 (10nM)-mediated ERK phosphorylation was completely abolished by PC7 (100nM), an inhibitor of PKR1, suggesting a selective action on PKR1 (S5 Fig).

### Cardioprotective effect of IS20 in a mouse model of myocardial infarction

**Fig 4:**
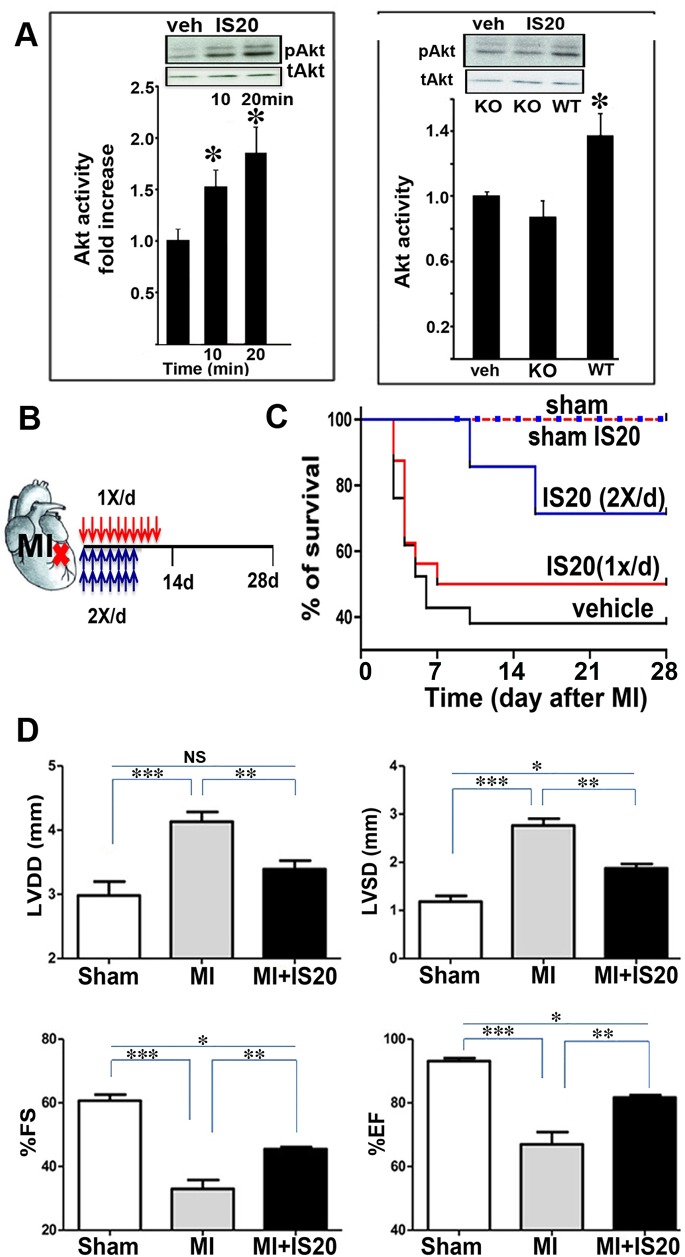
Evaluation of the activity and cardioprotective effects of the non-peptide PKR1 agonist IS20 *in vitro* and *in vivo*. **A.** IS20 increased Akt activation in hearts, 10 and 20 minutes after its i.p. (0.5mg/kg) injection in mice (*n* = 6). The activation of Akt by IS20 (0.5mg/kg, i.p.) was completely abolished in PKR1-KO mouse hearts. * shows p<0.05. (*n* = 4). **B.** Representative protocol for the treatment of mice with IS20 (i.p. 0.5mg/kg) after MI. **C.** Kaplan-Meier survival curve showing the increase in survival following the injection of IS20 once daily for 10 days or twice daily for seven days after MI (*n* = 20 each group). **D.** Echocardiographic analyses showing the larger left ventricular diastolic diameter (LVDD) and left ventricular systolic diameter (LVSD) in mice treated with IS20 than in vehicle-treated mice, after MI. Improvements in heart function, as illustrated by the changes in the ejection and shortening fractions in mice treated with IS20 after MI. Data are expressed as the mean ± SEM. * shows p<0.05 versus values from sham samples, **p<0.02 versus values from MI samples, ***p<0.01 versus values from sham samples by ANOVA test. NS: not significant.

As the aim of this study was to develop cardioprotective agents, we then examined the effects of IS20 on a key survival pathway in the heart: Akt activation. IS20 increased Akt activation by a factor of 1.8 in the heart 20 minutes after its injection into mice (i.p. 0.5mg/kg) (Fig 4A). To test *in vivo* the involvement of PKR1 in the IS20-mediated Akt activation, we performed the same experiment on the mice deficient for PKR1. Neither IS20 (0.5mg/kg i.p.) nor vehicle significantly modified Akt phosphorylation in PKR1-KO hearts (Fig 4A, right panel). These data indicate that IS20 selectively activates PKR1 to promote Akt phosphorylation *in vivo*.

The IS20 treatment protocols used after MI are shown in Fig 4B. The injection of IS20 once daily for 10 days after MI increased mouse survival by 13%. However, the treatment of mice twice daily with this molecule for seven days after MI strongly increased survival to 33% (Fig 4C). Echocardiography showed improvements in the shortening and ejection fractions in mice treated with IS20, 28 days after MI (Fig 4D). Note that vehicle or IS20 treated sham operated mice did not have any mortality.

**Fig 5:**
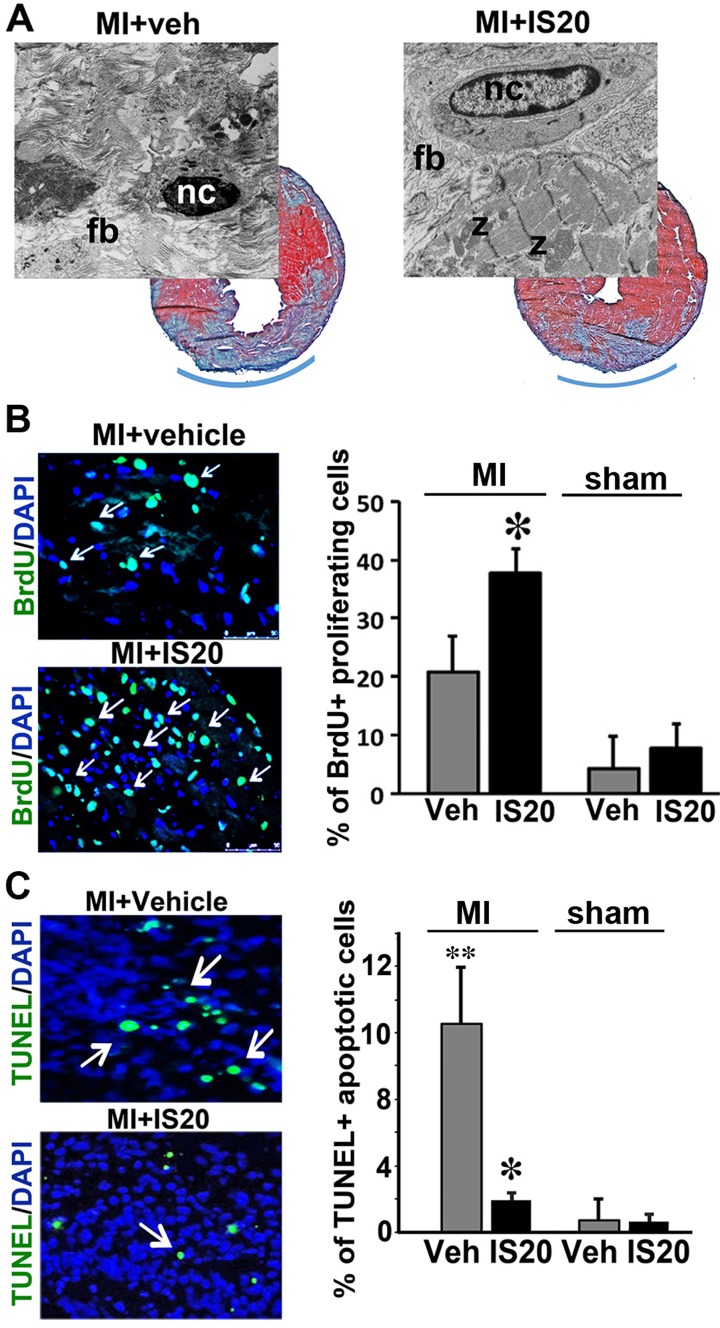
Proliferative and survival effect of non-peptide PKR1 agonist during cardiac remodeling. **A.** Malory staining of hearts treated with vehicle or IS20 (0.5mg/kg, i.p.), three days after MI. Blue staining indicates areas of scarring in the vehicle and IS20-treated hearts. Electron microscopy analyses revealed (in the indicated area) necrotic cardiomyocytes, and an accumulation of collagen fibers in the vehicle-treated hearts. By contrast, in the hearts of the IS20-treated animals, intact cardiomyocytes with sarcomeres (z bands) were observed in the zone bordering the scar area. **B.** Representative illustration for the BrdU staining of hearts 3 days after MI. The histogram shows a large increase in the number of BrdU-positive proliferating cells around the scar area following IS20 treatment. **C.** Representative illustration of TUNEL staining for hearts 3 days after MI. The histogram shows that IS20-treated hearts have far fewer TUNEL-positive apoptotic cells around the scar area. Data are expressed as the mean ± SEM. * shows p<0.05 versus values from MI vehicle treated samples by Student’s *t*-test.

We then investigated the mechanism underlying the cardioprotective effect of IS20 in the mouse model of MI. The vehicle- and IS20-treated mice were sacrificed three days after MI infarction or sham operation, and proliferation and apoptotic indices were determined for their hearts. The histological analyses of the MI hearts revealed a smaller area of scarring in the IS20-treated mice, as demonstrated by the size of the fibroblast zone stained blue with Malory stain (Fig 5A). Electron microscopy revealed severe collagen fiber accumulation and the presence of necrotic cardiomyocytes in the vehicle-treated MI heart sections. By contrast, in the IS20-treated MI hearts, cardiomyocytes in the border zone were still intact, with apparent Z bands. Less collagen was found to have accumulated, consistent with the results of Mallory staining. Consistently, three days after MI, IS20-treated hearts displayed lower rates of cardiomyocytes death, as determined in TUNEL assays (Fig 5C), and by co-staining of the heart sections with active caspase-3 and a cardiomyocytes marker troponin-I (S6A Fig). Tunel positive cell numbers were not altered in the sham-operated hearts treated with either IS20 or vehicle (S6B Fig). The adult hearts exhibited a few BrdU positive cells after sham operation treated with vehicle or IS20 (S6B Fig), whereas BrdU-positive cells significantly appears in the injured hearts (Fig 5B). However, in the IS20-treated damaged hearts proliferation rates were two times higher around the scar area as compare to vehicle-treated damaged hearts (Fig 5B).

**Fig 6:**
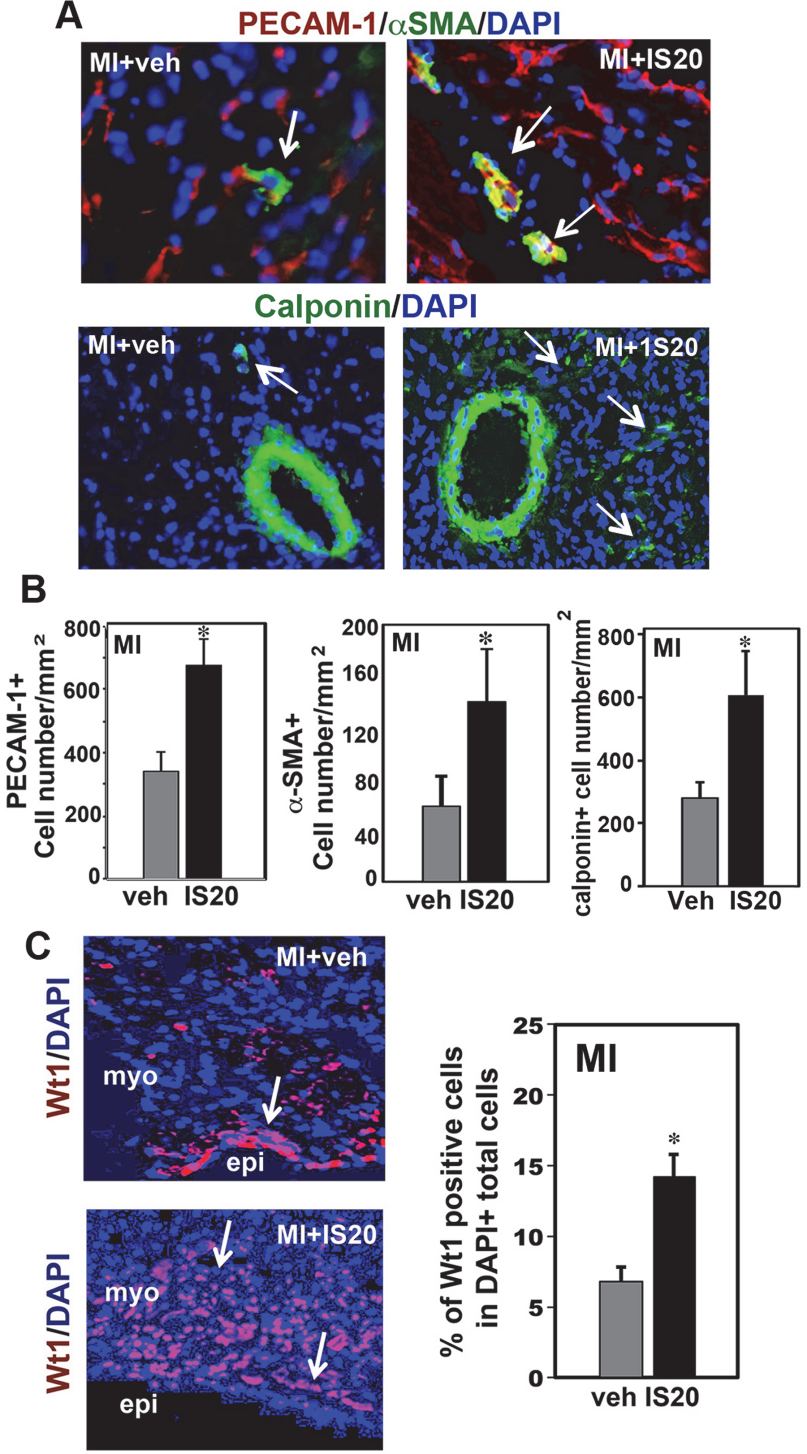
Increase neovascularization, capillary formation and cardiac progenitor cell numbers by non-peptide PKR1 agonist during cardiac remodeling. **A.** Representative Illustration of PECAM-1-positive endothelial cells and α-SMA- and calponin-positive smooth muscle cells in the vehicle- and IS20-treated hearts three days after MI. The nuclei of all cells are stained with DAPI. The histogram shows the larger number of capillary networks and vessels in IS20-treated hearts. **B.** Representative Illustration of Wt1 (progenitor cell marker)-positive cells in the vehicle- and IS20-treated hearts. IS20-treated hearts contained larger numbers of Wt1-positive epicardial progenitor cells than vehicle-treated hearts, three days after MI. Data are expressed as the mean ± SEM. * shows p<0.05 versus values from MI vehicle treated samples by Student’s *t*-test.

We then investigated vascularization in the heart. IS20-treated MI hearts had larger numbers of PECAM-1-positive endothelial cells in the border zone of the scar area (Fig 6A) as compared to vehicle-treated MI hearts. Immunostaining of the hearts with smooth muscle specific markers such as alpha-smooth muscle actin (α-SMA) and calponin (a marker of terminal smooth muscles) revealed an significantly elevated number of smooth muscle cells by IS20 treatment in the MI hearts. The adult hearts exhibited similar number of PECAM-1 and α-SMA positive cells after sham operation treated with vehicle or IS20 (S6 Fig). IS20-treated MI hearts were also found to have significantly larger numbers of Wt1-positive epicardial progenitor cells (Fig 6B). The adult hearts exhibited a few Wt1 positive cells after sham operation treated with vehicle or IS20, whereas Wt1-positive cells significantly appears in the injured hearts.

## Discussion

PKR1 signaling has recently been identified as a novel target for the treatment of heart failure, through the induction of cardiomyocyte survival and angiogenesis, and the promotion of progenitor cell expansion and differentiation for heart regeneration [6, 7, 9]. Therefore the development of non-peptide PKR1 agonists holds great promise for treating heart failure. We describe here the discovery of the first PKR1 agonists inducing cardiac regeneration after heart failure.

Adopting computational procedures, we identified an allosteric site in addition to the orthosteric sites in PKR1. The endogenous peptide ligand MIT and prokineticin-2 were found to bind to the orthosteric extracellular binding site of PKR1. Indeed, the binding of prokineticin-2 to the extracellular surface of PKR1 resembles the interaction of many peptides with their cognate GPCR [36]. Kallmann syndrome mutations affecting prokineticin receptors have highlighted the crucial role of ECL in prokineticin-2 binding [41]. However, the non-peptide ligands IS1 and IS20 did not compete with MIT, which has an amino-acid sequence 60% identical to that of prokineticin-2. The IS ligands bound to an allosteric transmembrane site to internalize the receptor.

Through docking studies, we identified the important residues interacting at the transmembrane site: Asn141^3.29^ Gln219, Arg144^3.32^, and Phe300^6.51^. These residues interacted in a specific manner with the IS20 ligand, and these interactions were found to be essential for agonistic activity. In particular, Arg144^3.32^ is analogous to Asp113^3.32^ and Phe300^6.51^ is analogous to Phe289^6.51^ of the β_2_-adrenergic receptor. Both these residues of the β_2_-adrenergic receptor have been identified as important sites for interactions with both agonists and antagonists. These positions have also been shown to be important for ligand binding in many other class A GPCRs and in other types of GPCRs, such as bitter taste receptors [42]. IS1 and IS20 interacted with PKR1 through a combination of cation-π and π-π interactions and hydrogen bonds with Asn141^3.29^, Arg144^3.32^, Gln219 and Phe300^6.51^. In addition, IS20 formed hydrogen bonds with with Tyr325^7.36^ (analogous to Ile309^7.36^ of the β_2_-adrenergic receptor) and interacted with Tyr123^2.65^ by a π-π stacking.

For class A GPCRs, many allosteric transmembrane binding sites have been described [43] [44]. For example, the endogenous peptide melanin-concentrating hormone binds to an orthosteric site on its receptor (MCHR). However, small-molecule MCHR antagonists bind to a transmembrane-bundle cavity of this receptor [45]. The mode of binding of IS1 and IS20 also differs considerably from that recently proposed for PKR1 antagonists [36]. Indeed, small-molecule antagonists bind PKR1 at the same allosteric binding site as IS compounds, but interact with Arg307^6.58^, Cys137^3.25^ and Glu119^2.60^, resulting in a totally different area of this pocket being occupied than that observed with IS compounds. The only residue targeted by both antagonists and IS compounds is Arg144^3.32^.

The allosteric modulation of GPCRs by a ligand has several key advantages over the traditional targeting of orthosteric sites [46]. The most important of these is that modulation by a ligand increases receptor-subtype selectivity, as observed for the IS compounds, which selectively activated PKR1, but not PKR2. Despite the similarity in the amino-acid sequences of the transmembrane-bundle antagonist binding sites between the two PKR subtypes, our study also shows that IS compounds can discriminate between PKR subtypes, indicating a high degree of PKR1 selectivity. In the presence of PKR2 or the absence of PKR1, IS compounds did not stimulate ERK activity. Moreover, CHO cells express more than 70 GPCRs involved in ERK kinase activation. Thus, our findings indicate that PKR1 agonists selectively activate the signaling through only one of these GPCRs, PKR1. The specificity of IS compounds for PKR1 was demonstrated *in vitro* and *in vivo*: i) neither IS1 or IS20 activated ERK in the absence of PKR1 in CHO cells, ii) IS compounds did not activate ERK in the cells expressing only PKR2, whereas prokineticin-2 a ligand for both receptors activated ERK in CHO-PKR2 cells, iii) IS20 was not able to activate Akt in PKR1-KO hearts where the PKR2 receptor is intact unlike total deletion of PKR1. It seems that IS20 acts as a positive allosteric modulator, since it elevates functional response of an orthosteric agonist (prokineticin-2), iv) the effect of IS20 was blocked by PC-7, a PKR1 antagonist.

We have previously shown that different PKR subtypes are coupled to different G proteins in endothelial cells. PKR1 is coupled to G_α11_ in endothelial cells and induces MAPK and PI3/Akt phosphorylation, promoting angiogenesis. By contrast, PKR2 is coupled to G_α12_ in endothelial cells, in which it internalizes G_α12_ and downregulates ZO-1 expression, leading to vacuole formation and the fenestration of these cells [10]. Thus, the allosteric modulation of PKR1 may also affect the interaction of this receptor with the various G proteins, and this may constitute an additional factor affecting PKR subtype specificity [46].

Through PKR1, prokineticin-2 activates several progenitor cells, including bone marrow-derived cells and EPDCs, to promote angiogenesis [47]. Through PKR1 signaling, it activates EPDCs, triggering the differentiation of endothelial and vascular smooth muscle cells, to promote neovasculogenesis [5,9]. This study provides the first proof-of-concept for the *in vivo* activity of non-peptide PKR1 agonists. The administration of IS20 in mice induced no signs of toxicity, such as diarrhea or weight loss (S7 Fig). Moreover, the i.p. administration of IS20 promoted Akt activity in the heart. Furthermore, IS20 decreased mortality and the size of the scar area and improved heart function after MI, by promoting proliferation, inhibiting apoptosis and increasing capillary formation. Consistent with our findings, the treatment of mice with Adv-PKR1 after MI has been shown to increase survival. The Adv-PKR1 treated mice had a smaller scar [6] area and larger numbers of Wt1-positive progenitor cells, with higher migration rates, in their hearts [7].

## Conclusion

These data clearly indicate that our non-peptide PKR1 agonists boost EPDC-mediated cardiac repair. Thus, this novel drug class may constitute a new generation of cardioprotective agents. Such compounds could also be useful for exploring the physiopathological roles of prokineticin and its receptors. PKR1 has also been implicated in the regulation of obesity [48] and diabetes [26]. Thus, the therapeutic potential of PKR1 agonist is currently investigated in mice models of obesity and diabetes in our laboratory.

## Supporting Information

S1 Fig Competitive binding and ERK activity assays. **A.** Binding competition between prokineticin-2 and ^125^I-MIT to PKR1 (IC_50_: 36pM), indicating the same binding site of PKR1 as MIT. IS1 does not replace ^125^I-MIT, verifying that it has an allosteric binding site with the same binding site of PKR1 as MIT. **B.** IS1 at 1 nM concentration enhances the functional response (ERK kinase activity) of endogenous ligand PK2 (10 nM) when the CHO-PKR1 cells were treated with these two ligands together, clearly indicating that IS1 acts as positive allosteric modulator.
(PDF)

S2 Fig Synthesis of IS1 analogues. (PDF)

S3 Fig Molecular properties for IS1 and IS20. The properties were calculated by means of QikProp (QikProp, version 2.2, Schrödinger, LLC, New York, NY, 2005).
(PDF)

S4 Fig Evaluation of the activity of the non-peptide PKR1 agonists IS1 and IS20 *in vitro*. **A.** IS20 (100 nM) cannot increase ERK activity in the presence of PKR2 in CHO cells. However, prokineticin-2 (10 nM) was able to activate ERK kinase via PKR2. **B.** IS20 acts as positive allosteric modulator by further increasing PK2 function on ERK activity. **C.** IS20 promotes Akt activity in a dose-dependent manner in CHO cells expressing PKR1 EC_50_ 10 nM). * p<0.05.
(PDF)

S5 Fig Evaluation of the PKR1 involvement by a PKR1 antagonist on IS20-mediated ERK activity. ERK activity induced by Prokineticin-2 (PK2, 10 nM) and IS20 (10 nM) were completely abolished by PC7, a PKR1 specific antagonist (100 nM). *p<0.05 compare to control, **p<0.05 compare to PC7 alone.
(PDF)

S6 Fig Detection of apoptosis and proliferation and vascularization in sham or MI hearts. **A.** Detection of cardiomyocytes death by active caspase-3 staining on heart sections. Illustration shows troponin positive (red) cardiomyocytes in caspase-3 positive (green) apoptotic cell population. Histogram shows the quantification of troponin+ cardiomyocytes in caspase-3 positive apoptotic cell population. *p<0.05). **B.** Representative illustration of Tunel (upper) and BrdU (lower) positive cells in the hearts of sham operated mice treated with vehicle or IS20, showing no differences between the groups (quantification was shown in Fig 4). **C.** Representative illustration of PECAM-1 and α-SMA positive cells in the hearts of sham operated mice treated with vehicle or IS20, showing no differences between the groups.
(PDF)

S7 Fig Body weight of mice treated with IS20 for 0.5 mg/kg. IS20 and its vehicle were injected i.p. to male and female mice (9 weeks old, *n* = 10 each). The body weight was measured every day for 40 days. No sign of toxicity was detected.
(PDF)

S1 File Supplementary experimental procedure. **Computational Details**
Homology ModelingHigh Throughput Docking (HTD)ADME+T properties prediction
(PDF)
