# Supplementary material for: Discovery and Cardioprotective Effects of the First Non-Peptide Agonists of the G Protein-Coupled Prokineticin Receptor-1
Source: PLoS One. 2015 Apr 1;10(4):e0121027. doi: 10.1371/journal.pone.0121027 (PMC4382091; doi:10.1371/journal.pone.0121027)
Supplement: S1 Fig — A. Binding competition between prokineticin-2 and 125I-MIT to PKR1 (IC50: 36pM), indicating the same binding site of PKR1 as MIT. IS1 does not replace 125I-MIT, verifying that it has an allosteric binding site with the same binding site of PKR1 as MIT. B. IS1 at 1 nM concentration enhances the functional response (ERK kinase activity) of endogenous ligand PK2 (10 nM) when the CHO-PKR1 cells were treated with these two ligands together, clearly indicating that IS1 acts as positive allosteric modulator. (PDF) [file pone.0121027.s001.pdf]

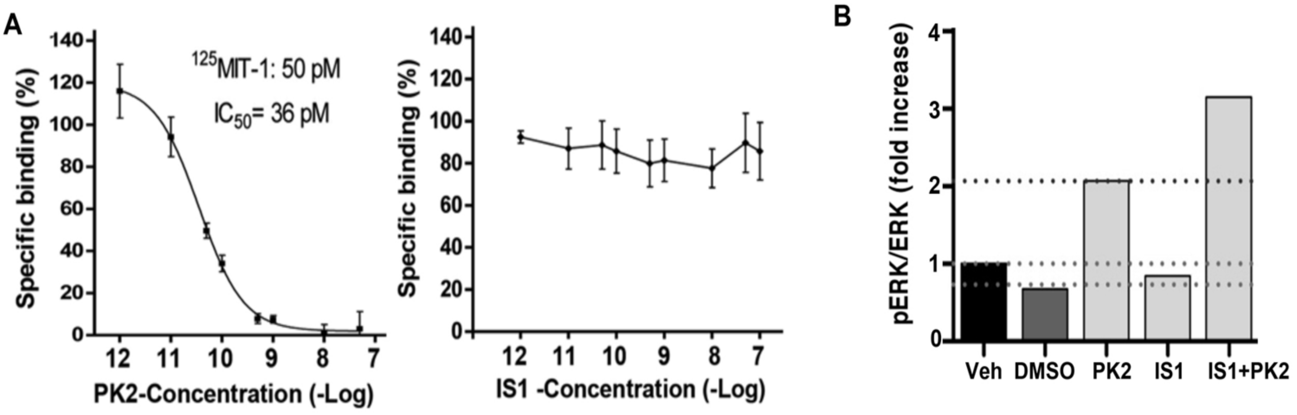

S1 Fig. Competitive binding and ERK activity assays: A. Binding competition between prokineticin-2 and ^{125}I-MIT to PKR1 (IC_{50}: 36pM), indicating the same binding site of PKR1 as MIT. IS1 does not replace ^{125}I-MIT, verifying that it has an allosteric binding site with the same binding site of PKR1 as MIT. **B.** IS1 at 1 nM concentration enhances the functional response (ERK kinase activity) of endogenous ligand PK2 (10nM) when the CHO-PKR1 cells were treated with these two ligands together, clearly indicating that IS1 acts as positive allosteric modulator.
