# Supplementary figures and images for: Discovery and Cardioprotective Effects of the First Non-Peptide Agonists of the G Protein-Coupled Prokineticin Receptor-1
Source: PLoS One. 2015 Apr 1;10(4):e0121027. doi: 10.1371/journal.pone.0121027 (PMC4382091; doi:10.1371/journal.pone.0121027)

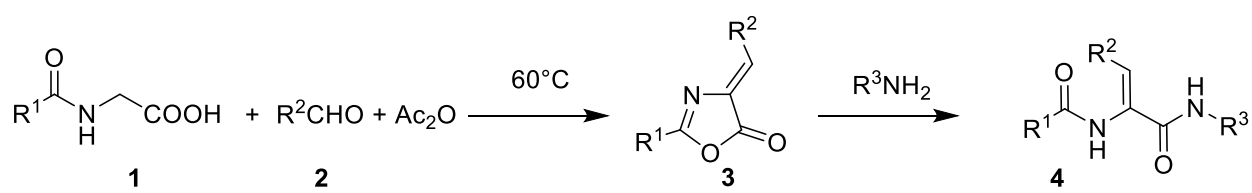

**S2 Fig. Synthesis of IS1 analogues.**

Supplement: S2 Fig — (PDF) [file pone.0121027.s002.pdf]
