# Supplementary material for: Discovery and Cardioprotective Effects of the First Non-Peptide Agonists of the G Protein-Coupled Prokineticin Receptor-1
Source: PLoS One. 2015 Apr 1;10(4):e0121027. doi: 10.1371/journal.pone.0121027 (PMC4382091; doi:10.1371/journal.pone.0121027)
Supplement: S3 Fig — The properties were calculated by means of QikProp (QikProp, version 2.2, Schrödinger, LLC, New York, NY, 2005). (PDF) [file pone.0121027.s003.pdf]

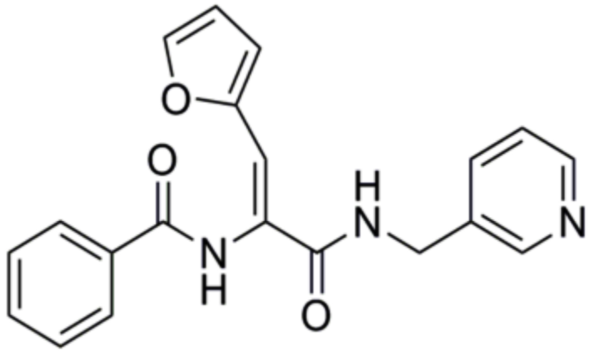

title: IS1
mol MW: 347.373
QPlogPo/w: 3.586
QPlogS: -4.577
PSA: 90.852
SASA: 643.152
volume: 1126.282
QPlogBB: -0.862
QPPCaco: 1121.426
QPPMDCK: 559.946
QPlogHERG: -6.863
QPpolrz: 39.073
QPlogKhsa: 0.159
CNS: -1
PercentHumanOralAbsorption: 100.0
ACxDN^.5/SA: 0.010865
IP(eV): 9.059
EA(eV): 0.525
glob: 0.813984
#NandO: 6
#amine: 0
#amide: 0
#acid: 0
#rtvFG: 1
#rotor: 7
#metab: 4
RuleOfFive: 0

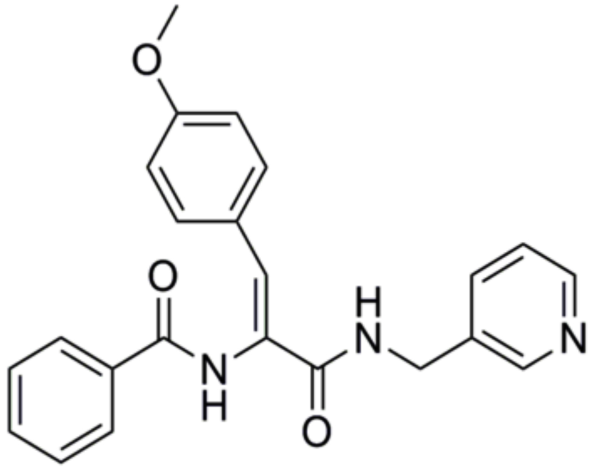

title: IS20
mol MW: 387.437
QPlogPo/w: 4.56
QPlogS: -6.436
PSA: 82.732
SASA: 763.888
volume: 1299.793
QPlogBB: -1.119
QPPCaco: 1059.964
QPPMDCK: 526.85
QPlogHERG: -8.004
QPpolrz: 45.718
QPlogKhsa: 0.495
CNS: -2
PercentHumanOralAbsorption: 100.0
ACxDN^.5/SA: 0.009513
IP(eV): 8.539
EA(eV): 0.808
glob: 0.754024
#NandO: 6
#amine: 0
#amide: 0
#acid: 0
#rtvFG: 1
#rotor: 8
#metab: 4
RuleOfFive: 0

S3 Fig. Molecular properties for IS1 and IS20. The properties were calculated by means of QikProp (QikProp, version 2.2, Schrödinger, LLC, New York, NY, 2005).
