# Supplementary material for: Discovery and Cardioprotective Effects of the First Non-Peptide Agonists of the G Protein-Coupled Prokineticin Receptor-1
Source: PLoS One. 2015 Apr 1;10(4):e0121027. doi: 10.1371/journal.pone.0121027 (PMC4382091; doi:10.1371/journal.pone.0121027)
Supplement: S4 Fig — A. IS20 (100 nM) cannot increase ERK activity in the presence of PKR2 in CHO cells. However, prokineticin-2 (10 nM) was able to activate ERK kinase via PKR2. B. IS20 acts as positive allosteric modulator by further increasing PK2 function on ERK activity. C. IS20 promotes Akt activity in a dose-dependent manner in CHO cells expressing PKR1 EC50 10 nM). * p<0.05. (PDF) [file pone.0121027.s004.pdf]

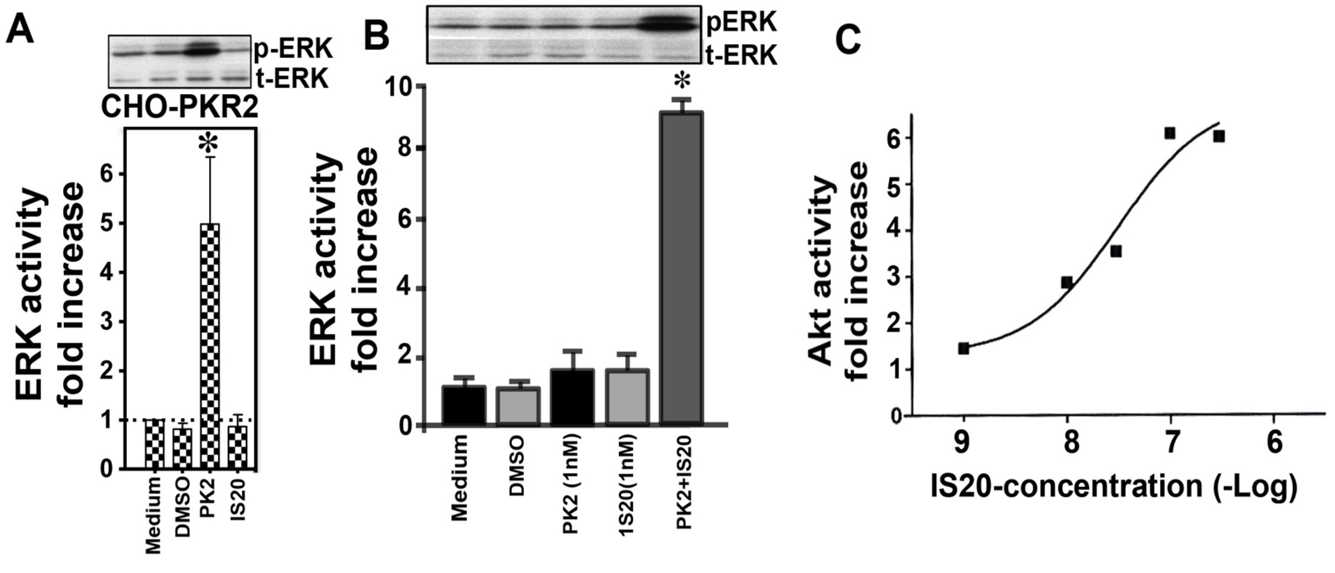

S4 Fig. Evaluation of the activity of the non-peptide PKR1 agonists IS1 and IS20 *in vitro*. A. IS20 (100 nM) cannot increase ERK activity in the presence of PKR2 in CHO cells. However, prokineticin-2 (10nM) was able to activate ERK kinase via PKR2. **B.** IS20 acts as positive allosteric modulator by further increasing PK2 function on ERK activity. **C.** IS20 promotes Akt activity in a dose-dependent manner in CHO cells expressing PKR1 EC_{50} 10nM). * $p<0.05$.
