# Supplementary material for: Discovery and Cardioprotective Effects of the First Non-Peptide Agonists of the G Protein-Coupled Prokineticin Receptor-1
Source: PLoS One. 2015 Apr 1;10(4):e0121027. doi: 10.1371/journal.pone.0121027 (PMC4382091; doi:10.1371/journal.pone.0121027)
Supplement: S5 Fig — ERK activity induced by Prokineticin-2 (PK2, 10 nM) and IS20 (10 nM) were completely abolished by PC7, a PKR1 specific antagonist (100 nM). *p<0.05 compare to control, **p<0.05 compare to PC7 alone. (PDF) [file pone.0121027.s005.pdf]

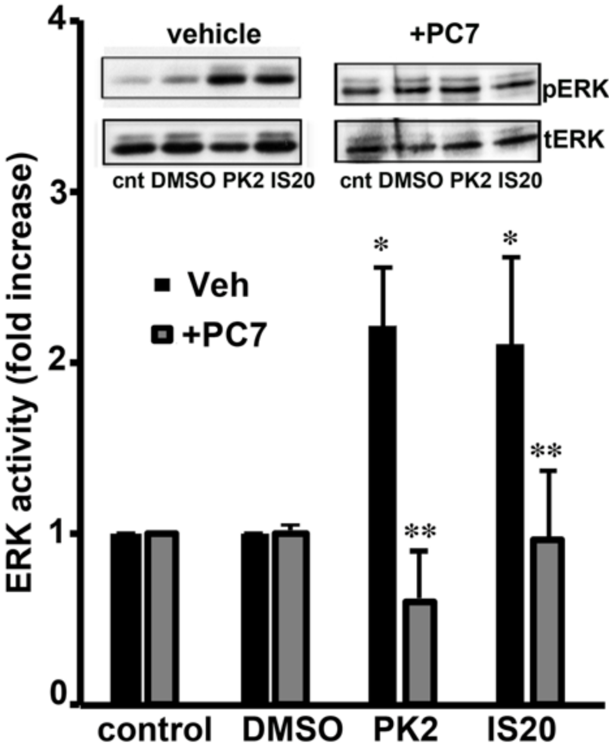

S5 Fig. Evaluation of the PKR1 involvement by a PKR1 antagonist on IS20-mediated ERK activity. ERK activity induced by Prokineticin-2 (PK2, 10nM) and IS20 (10nM) were completely abolished by PC7, a PRK1 specific antagonist (100 nM). * $p<0.05$ compare to control, ** $p<0.05$ compare to PC7 alone.
