# Supplementary material for: Discovery and Cardioprotective Effects of the First Non-Peptide Agonists of the G Protein-Coupled Prokineticin Receptor-1
Source: PLoS One. 2015 Apr 1;10(4):e0121027. doi: 10.1371/journal.pone.0121027 (PMC4382091; doi:10.1371/journal.pone.0121027)
Supplement: S6 Fig — A. Detection of cardiomyocytes death by active caspase-3 staining on heart sections. Illustration shows troponin positive (red) cardiomyocytes in caspase-3 positive (green) apoptotic cell population. Histogram shows the quantification of troponin+ cardiomyocytes in caspase-3 positive apoptotic cell population. *p<0.05). B. Representative illustration of Tunel (upper) and BrdU (lower) positive cells in the hearts of sham operated mice treated with vehicle or IS20, showing no differences between the groups (quantification was shown in Fig 4). C. Representative illustration of PECAM-1 and α-SMA positive cells in the hearts of sham operated mice treated with vehicle or IS20, showing no differences between the groups. (PDF) [file pone.0121027.s006.pdf]

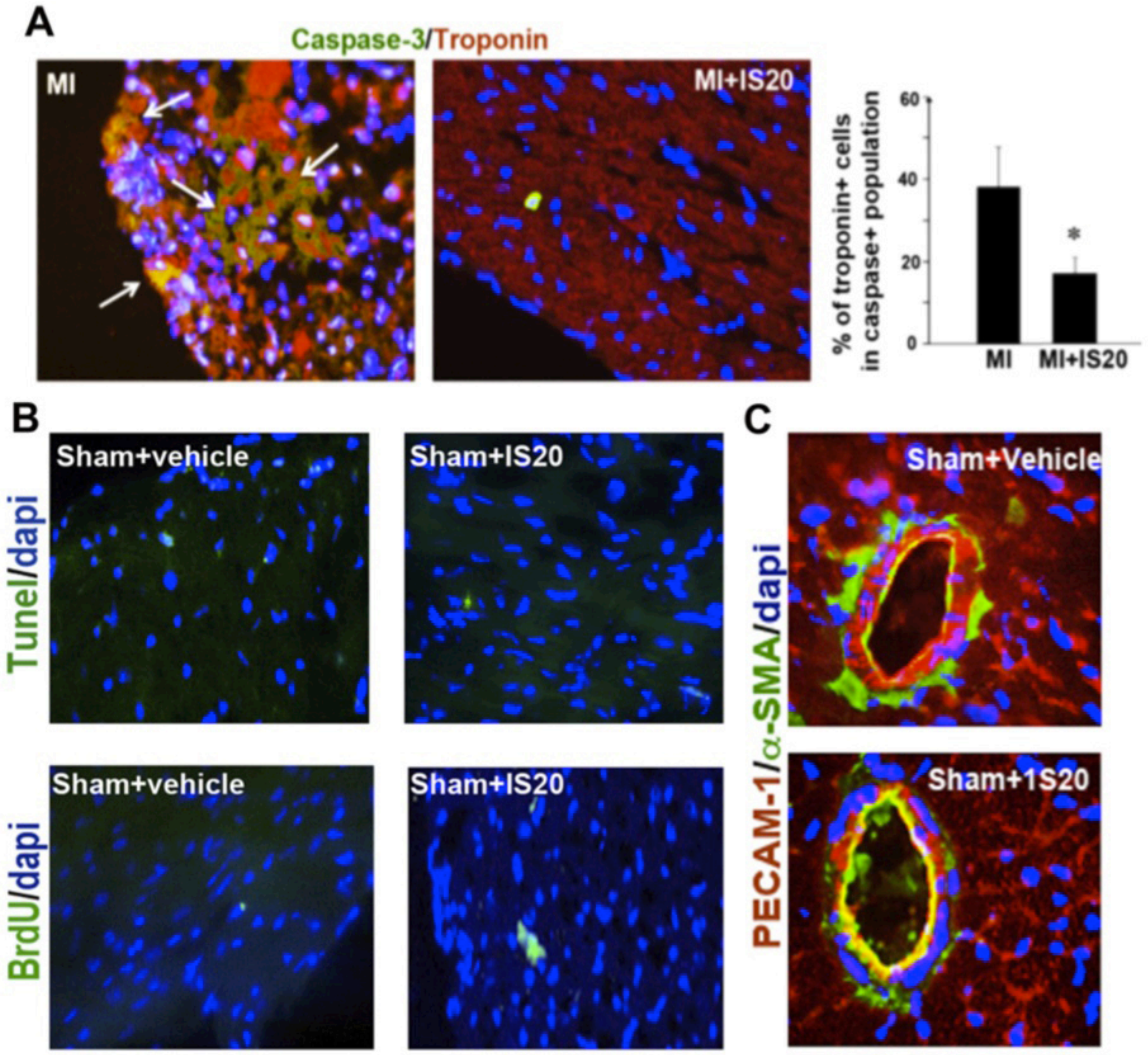

S6 Fig. Detection of apoptosis and proliferation ans vascularization in sham or MI hearts.

A. Detection of cardiomyocytes death by active caspase-3 staining on heart sections. Illustration shows troponin positive (red) cardiomyocytes in caspase-3 positive (green) apoptotic cell population. Histogram shows the quantification of troponin+ cardiomyocytes in caspase-3 positive apoptotic cell population. * p<0.05). **B.** Representative illustration of Tunel (upper) and BrdU (lower) positive cells in the hearts of sham operated mice treated with vehicle or IS20, showing no differences between the groups (quantification was shown in figure 4). **C.** Representative illustration of PECAM-1 and alpha-SMA positive cells in the hearts of sham operated mice treated with vehicle or IS20, showing no differences between the groups.
