# Supplementary material for: Discovery and Cardioprotective Effects of the First Non-Peptide Agonists of the G Protein-Coupled Prokineticin Receptor-1
Source: PLoS One. 2015 Apr 1;10(4):e0121027. doi: 10.1371/journal.pone.0121027 (PMC4382091; doi:10.1371/journal.pone.0121027)
Supplement: S7 Fig — IS20 and its vehicle were injected i.p. to male and female mice (9 weeks old, n = 10 each). The body weight was measured every day for 40 days. No sign of toxicity was detected. (PDF) [file pone.0121027.s007.pdf]

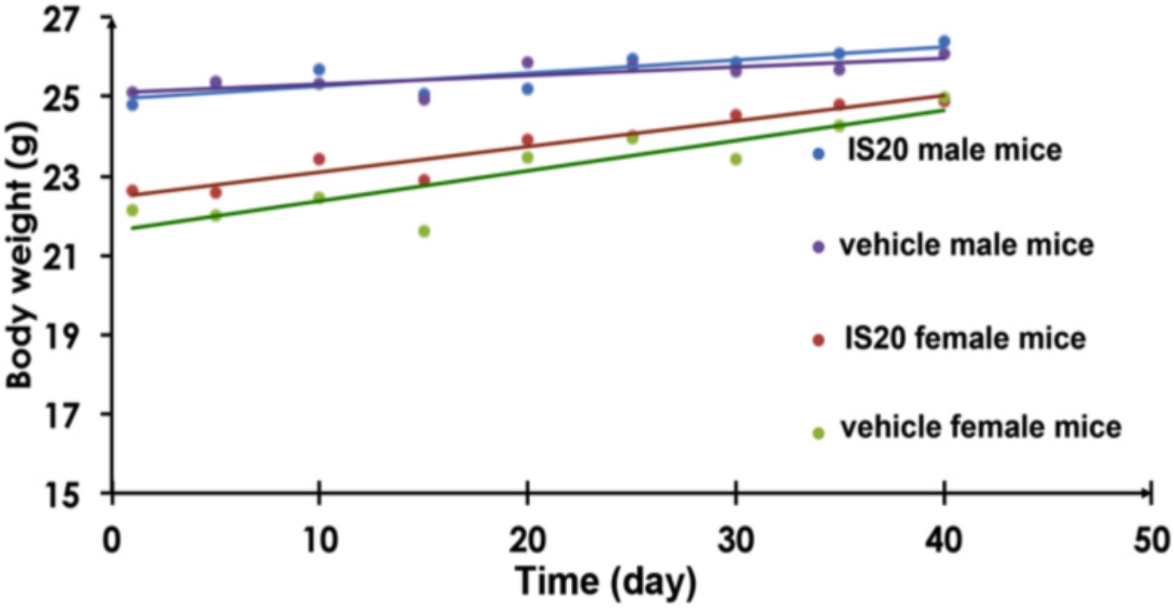

S7 Fig. Body weight of mice treated with IS20 for 0.5 mg/kg. IS20 and its vehicle were injected i.p. to male and female mice (9 weeks old, n=10 each). The body weight was measured every day for 40 days. No sign of toxicity was detected.
