# Supplementary material for: Discovery and Cardioprotective Effects of the First Non-Peptide Agonists of the G Protein-Coupled Prokineticin Receptor-1
Source: PLoS One. 2015 Apr 1;10(4):e0121027. doi: 10.1371/journal.pone.0121027 (PMC4382091; doi:10.1371/journal.pone.0121027)

Discovery and cardioprotective effects of the first non-peptide agonists of the G protein-coupled prokineticin receptor -1

Adeline Gasser,[1‡] Simone Brogi,[2,3‡] Kyoji Urayama,[1,4] Toshishide Nishi,[1,4] Hitoshi Kurose,[4] Andrea Tafi,[3] Nigel Ribeiro,[5] Laurent Désaubry[5*] and Canan G. Nebigil[1*]

Supplementary experimental procedure

Computational Details

a) Homology Modeling

Sequence alignment and protein folding was performed by means of CPHmoldels 2.0. The protein sequence of human PKR1 (entry Q8TCW9, 393 aa) was taken in fasta format from UniprotKB. Subsequently, the protein sequence was used as input in the CPHmodels web interface, and was utilized from the server as a query for finding structural templates. Once the best template has been found, Cα-atom coordinates are extracted according to the sequence alignment and used as a starting point for the homology-modeling process. Missing atoms were added and the structure was refined. The crystal structure of turkey β1 adrenergic receptor (PDB ID:2VT4) was used as a template for model building (sequence identity 29.4%; sequence similarity 41%). Although this value is not very high, it is similar to cases in which GPCRs modeling has been successfully applied in ligands identification [45]. The obtained model was submitted to extensive refinement protocol in Prime environment. In particular, we have performed a side-chains optimization and loops refinement by Prime (Prime version 1.7, Schrödinger, LLC, New York, NY, 2005) using ultra extended in serial loop sampling options as recommended by means of Prime user manual for loops with 10 or more residues. Further structure optimization was carried out by MacroModel (MacroModel, version 9.1; Schrodinger, LLC: New York, 2005) application implemented in Maestro using the Optimized Potentials for Liquid Simulations-all atom (OPLS-AA) force field 2005[46]. The solvent effects were simulated using the analytical Generalized-Born/Surface-Area (GB/SA) model[47] and no cutoff for non-bonded interactions was selected. Polak-Ribierè Conjugate Gradient (PRCG) method with 100,000 maximum iterations and 0.001 gradient convergence threshold was employed. Moreover, the minimized PKR1 homology model was submitted to Protein Preparation Wizard Workflow (Schrodinger. Docs & Known Issues. Protein Preparation Wizard. http://www.schrodinger.com/supportdocs/18/16). This protocol allowed us to obtain a reasonable starting structure of the receptor for our molecular docking calculations, by a series of computational steps. In particular, we performed three steps in order to: i) add missed hydrogens; ii) optimize the orientation of hydroxyl groups, Asn, and Gln, and the protonation state of His; and iii) perform a constrained refinement with the impref utility, setting the max root-mean-square deviation (RMSD) of 0.30 Å. Moreover, by using Protein Preparation Wizard Workflow we have taken into account also the potential formation of disulfide bonds. In fact, during the optimization we have chosen “create disulfide bonds”. Following this method, if two cysteine residues are at appropriate distance the application is able to form the disulfide bonds. In particular, we have found a disulfide bonds highly conserved among β-adrenoreceptors and PKR1. Accordingly, a disulfide bond that connects ECL2 to TM3 was found in the β_1-adrenergic (2VT4) (that was used as template; cys114-cys199) and β_2-adrenergic receptor (2RH1; cys106-190); since these residues appear to be conserved also in PKR1 (cys137-cys217) a disulfide bond between ECL2 and TM3 is considered in our model. Moreover a minimization by using impref utility was performed. This latter consists of a cycles of energy minimizations based on the impact molecular mechanics engine and on the OPLS_2005 force field.

The modeled protein was validated by Ramachandran plot generated by Maestro suite. In the final model, 85.8% of residues corresponding to 254 amino acids are in most favorable region, 35 amino acids (11.85% of residues) are in additional allowed regions of the Ramachandran plot. Only 7 amino acids (2.35%) are in disallowed regions of the plot. The results revealed that over 97% of the residues of our PKR1 refined model sit in the allowed regions of Ramachandran Plot. This value is more than the cut-off value (96.1%) defined for the most reliable models (Lüthy R. *et al.* (1994) Protein Sci.3: 139-46). Consequently, the stereo chemical quality of our PKR1 homology model was found acceptable displaying a very low percentage of residues having phi/psi angles in outlier region.

The ICMPocketFinder application was used to identify the binding pocket in the PKR1 homology model. The algorithm builds a grid map of a binding potential, and the position and size of the ligand-binding pocket are determined based on the construction of equipotential surfaces along those maps. Notably, where applicable, Ballesteros-Weinstein designations was calculated by using Maestro python script "GPCR generic number" and it appears as superscript. Moreover, the analogous residues between PKR1 and β-adrenergic receptor were established by superposition of the GPCRs performed by PyMOL

b) High Throughput Docking (HTD)

High Throughput Docking was carried out using GOLD 3.0.1 (Genetic Optimization for Ligand Docking) software from Cambridge Crystallographic Data Center, U.K. that uses the Genetic algorithm (GA), running under Linux OS. This method allows a partial flexibility of protein and full flexibility of ligand. For each of the 100 independent GA runs, a maximum number of 100000 GA operations were performed. The active site radius of 10 Å was chosen by XYZ coordinates from the center of the predicted binding site in the homology model of PKR1. Default cutoff values of 2.5 Å (dH-X) for hydrogen bonds and 4.0 Å for van der Waals distance were employed. In order to rank the docked compounds from Asinex Gold Collection database (Asinex Ltd., Russia) the fitness function ChemScore was evaluated. The database was prepared by means of Maestro in order to minimize the existent structures by the application of multiple minimization method available in Macromodel (MacroModel, version 9.1; Schrodinger, LLC: New York, 2005). This latter coupled to a visual inspection was used for excluding redundant and corrupted strucutres. The selection of compounds employing HTD protocol was based on the use of a ChemScore cutoff of 40 coupled with cluster analysis and visual inspection. Following this protocol 10 top-ranked compounds were chosen based on unique cluster of docked solutions. Moreover, for IS1 and IS20 the original poses were rescored by using Goldscore scoring function, confirming the high reliability of the proposed binding modes.

c) ADME+T properties prediction

QikProp generates physically relevant descriptors, and uses them to perform ADME+T predictions. The computed ADME+T properties for selected compounds are reported in Figure S1. The calculated properties are (range or recommended value for 95% of known drugs described in QikProp user manual are reported in brackets):
- QPlogP predicted octanol/water partition coefficient (-2 – 6.5);
- QPlogS predicted aqueous solubility in mol/dm^3 (-6.5 – 0.5);
- PSA Van der Waals surface area of polar nitrogen and oxygen atoms (7.0 – 200.0);
- SASA total solvent accessible surface area (SASA) in square angstroms using a probe with a 1.4 Å radius (300.0 – 1000.0);
- Volume total solvent-accessible volume in cubic angstroms using a probe with a 1.4 Å radius (range or recommended value for 95%of known drugs 500.0 – 2000.0);
- QPlogBB predicted brain/blood partition coefficient (range or recommended value for 95%of known drugs -3 – 1.2);
- QPPCaco predicted apparent Caco-2 cell permeability in nm/sec (<25 poor >500 great);
- QPPMDCK predicted apparent MDCK cell permeability in nm/sec (<25 poor >500 great);

- QPlogHERG predicted IC_{50} value for blockage of HERG K^+ channels (below -5);
- QPpolrz predicted polarizability in cubic angstroms. (13.0 – 70.0);
- QPlogKhsa prediction of binding to human serum albumin. (-1.5 – 1,5);
- CNS predicted central nervous system activity on a –2 (inactive) to +2 (active) scale;
- ACxDN^.5/SA Index of cohesive interaction in solids. This term represents the relationships (*accptHB*(*donorHB*$^{1/2}$))/(*SA*). (0.0 – 0.05);
- IP(eV) PM3 calculated ionization potential (7.9 – 10.5);
- EA(eV) PM3 calculated electron affinity (-0.9 – 1.7);

glob globularity descriptor, (4π r^2)/(SASA) where r is the radius of a sphere with a volume equal to the molecular volume. Globularity is 1.0 for a spherical molecule (range or recommended value for 95% of known drugs 0.75 – 0.95);

- #NandO number of nitrogen and oxygen atoms (2 – 15);
- #amine number of non-conjugated amine groups (0 – 1);
- #amide number of non-conjugated amide groups (0 – 1);
- **#**acid number of carboxylic acid groups (0 – 1);
- #rtvFG number of reactive functional groups; the specific groups are listed in the *jobname* .out file. The presence of these groups can lead to false positives in HTS assays and to decomposition, reactivity, or toxicity problems *in vivo* (0 – 2);
- #rotor number of non-trivial (not CX3), non-hindered (not alkene, amide, small ring) rotatable bonds (0 – 15);
- #metab Number of likely metabolic reactions (1 – 8);
- RuleOfFive indicates the number of violation of Lipinski's rule of five. Compounds that satisfy these rules are considered drug-like (maximum is 4).

General procedure for the synthesis of IS compounds:

An amine (4 mmol) and an azlactone (4 mmol) in suspension in CH_2Cl_2/EtOH (8 mL, 1/1) was sonicated for 10 min. After stirring overnight at rt, the solvent was removed under vacuum, and the residue was purified by flash chromatography (EtOAc to EtOAc/EtOH, 80/20) to give the expected product a white solid (purity > 97%). The purity of the synthesized compounds was over 95% based on reversed-phase HPLC analyses (Hypersil Gold column 30×1 mm, C18) under the following conditions: flow rate: 0.3 mL/min; buffer A: CH_3CN, buffer B: 0.01% aqueous TFA; gradient: 98-10% buffer B over 8 min.

Mass spectrophotometer analysis showed that IS20 are soluble in cell culture medium up to 20 micromolar

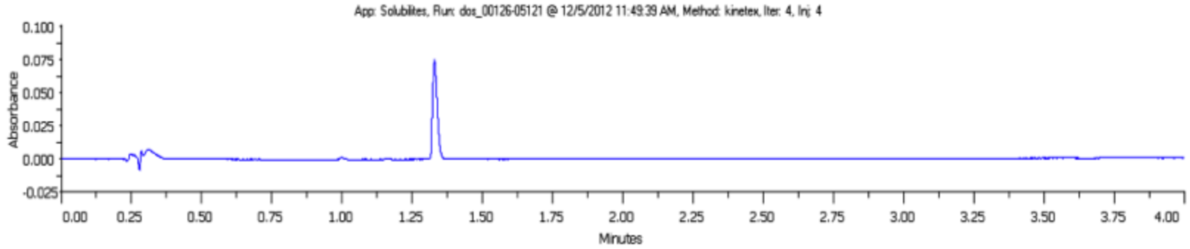

Supplement: S1 File — Computational Details Homology ModelingHigh Throughput Docking (HTD)ADME+T properties prediction (PDF) [file pone.0121027.s008.pdf]
